# Identification of an Essential Region for Translocation of *Clostridium difficile* Toxin B

**DOI:** 10.3390/toxins8080241

**Published:** 2016-08-15

**Authors:** Shuyi Chen, Haiying Wang, Huawei Gu, Chunli Sun, Shan Li, Hanping Feng, Jufang Wang

**Affiliations:** 1 School of Bioscience and Bioengineering, South China University of Technology, Guangzhou 510006, China; shuyichan@foxmail.com (S.C.); yingzi224926@163.com (H.W.); guhuawei1990@163.com (H.G.); chunlis@163.com (C.S.); lishan@scut.edu.cn (S.L.); 2 Department of Microbial Pathogenesis, University of Maryland Dental School, Baltimore, MD 21201, USA; hfeng@umaryland.edu

**Keywords:** *Clostridium difficile*, toxin, translocation, endocytosis, conformational change

## Abstract

*Clostridium difficile* toxin A (TcdA) and toxin B (TcdB) are the major virulence factors involved in *C. difficile*-associated diarrhea and pseudomembranous colitis. TcdA and TcdB both contain at least four distinct domains: the glucosyltransferase domain, cysteine protease domain, receptor binding domain, and translocation domain. Few studies have investigated the translocation domain and its mechanism of action. Recently, it was demonstrated that a segment of 97 amino acids (AA 1756–1852, designated D97) within the translocation domain of TcdB is essential for the in vitro and in vivo toxicity of TcdB. However, the mechanism by which D97 regulates the action of TcdB in host cells and the important amino acids within this region are unknown. In this study, we discovered that a smaller fragment, amino acids 1756–1780, located in the *N*-terminus of the D97 fragment, is essential for translocation of the effector glucosyltransferase domain into the host cytosol. A sequence of 25AA within D97 is predicted to form an alpha helical structure and is the critical part of D97. The deletion mutant TcdB_∆1756–1780_ showed similar glucosyltransferase and cysteine protease activity, cellular binding, and pore formation to wild type TcdB, but it failed to induce the glucosylation of Rho GTPase Rac1 of host cells. Moreover, we found that TcdB_∆1756–1780_ was rapidly degraded in the endosome of target cells, and therefore its intact glucosyltransferase domain was unable to translocate efficiently into host cytosol. Our finding provides an insight into the molecular mechanisms of action of TcdB in the intoxication of host cells.

## 1. Introduction

*Clostridium difficile* is the major cause of antibiotic-associated diarrhea and pseudomembranous colitis worldwide. Two exotoxins, toxin A (TcdA) and toxin B (TcdB), are the major virulence factors involved in *C. difficile* infection (CDI) [1,2], and both belong to the family of clostridial glucosylating toxins. The toxins are multi-domain proteins containing at least four functional domains [3]. The *N*-terminus of the toxin harbors the glucosyltransferase domain (GTD) that inactivates host Rho GTPases by glucosylation [4,5] and a cysteine protease domain (CPD) responsible for autoprocessing [6,7,8]. The *C*-terminus, consisting of combined repetitive oligopeptides (CROP), is predicted to be a receptor binding domain (RBD) [9,10]. The receptor for TcdB has been identified recently [11,12], but additional receptors may exist [13,14]. A large region between the CPD and RBD is thought to be the translocation domain (TD) which is important for delivery of *N*-terminal enzymatic domains into the host cytosol via pore formation [15,16,17,18,19].

The molecular mode of action of the toxins is not completely understood, but it is widely accepted that the toxins bind to cell surface receptors via the RBD, and then enter the cells through endocytosis to reach endosomal compartments, in which the toxins undergo conformational change [18] and perform pore formation [15,16] and eventually deliver the GTD across the endosomal membrane into the cytosol [20]. However, until now the structure of the TD and the molecular mechanism of toxin translocation have not been clarified. In recent years, much work has been done to explore the structure-function relationship of the translocation domain. In 1992, Von Eichel-Streiber et al. [10] found a hydrophobic area (amino acids 956–1128) which is involved in the membrane insertion of TcdB. Qa’Dan M. et al. [18] reported in 2000 that the low pH of early endosomes induces conformational changes of TcdB, resulting in exposure of the hydrophobic area and membrane insertion. Torsten Giesemann et al. [21] showed in 2006 that TcdA induces pore formation in an acidic environment in colonic cells that depends on the presence of cholesterol. In 2011, Selda Genisyuerek and coworkers [16] found a region between amino acids 830–990 that is essential for pore formation of TcdB. Last year, Zhifen Zhang and coworkers [22] uncovered the highly sensitive residues located between amino acids 1035–1107 that are important for TcdB pore formation and transloction.

Recently, it was demonstrated that the D97 fragment is essential for the toxicity of TcdB [23]. Deletion of the D97 fragment does not influence the toxin’s glucosyltransferase, cysteine protease activity, and endocytosis, but leads to failure in delivery of the GTD into host cytosol. It was hypothesized that the D97 fragment is essential for translocation of TcdB because, after its deletion, toxin molecules were trapped in the endosome.

This study aimed to find the critical structure in the D97 fragment and provide insights into its function. The region of AA 1756–1780 within the D97 fragment was determined to be essential for the translocation of TcdB.

## 2. Results

### 2.1. Prediction of the Secondary Structure of the D97 Segment

**Figure 1:**
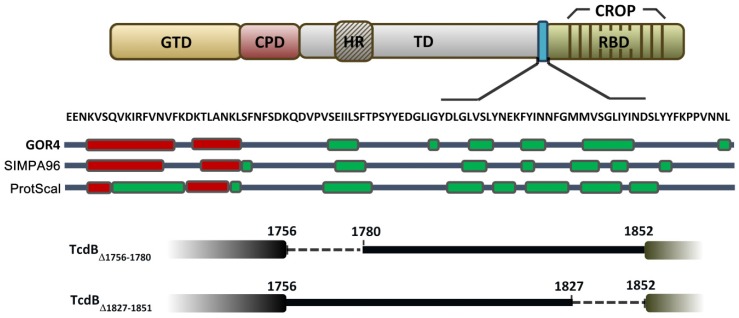
Structural diagram of TcdB_fl_, TcdB_∆1756–1780_, and TcdB_∆1827–1851_ showing the domain structure of TcdB and the alpha helix, which is located in AA 1756–1780. GTD harbors glucosyltransferase activity. CPD can induce autocleavage of the toxin in the presence of InsP_6_. TD is responsible for GTD domain delivery. RBD is involved in cellular binding and endocytosis. The secondary structure of D97 was predicted using three algorithms (GOR4, SIMPA96, Chou-Fasman (ProtScal)). Alpha-helical and beta-sheet structures are presented as red and green boxes, respectively. The mutant toxins TcdB_∆1756–1780_ and TcdB_∆1827–1851_ with AA 1756–1780 and 1827–1851 deleted, respectively, are shown in the figure. Abbreviations: GTD, glucosytransferase domain; CPD, cysteine protease domain; TD, translocation domain; HR, hydrophobic region within the translocation domain; RBD, receptor binding domain; D97, the region AA 1827-1851 of TcdB.

To gain insight into the structure-function relationship of TcdB, the secondary structure of the D97 segment was predicted by the algorithms GOR4, SIMPA96, and Chou-Fasman (ProtScal), which showed that the region of AA 1756–1780 of TcdB putatively formed an alpha-helical structure (Figure 1). There is no obvious alpha helical structure formation within the region of AA 1781–1851 according to the predictions. Therefore, we hypothesized that the AA 1756–1780 region of D97 is important. Therefore, 25AA (1756–1780) were deleted to construct the deletion mutant TcdB_∆1756–1780_ (Figure 1). The deletion mutant TcdB_∆1827–1851_ (Figure 1), with deletion of a different 25AA, 1827–1851, located in the *C*-terminus of D97, served as the control in the following experiments. The deletion mutants were expressed with His_6_-tags at the *C*-terminal in a *B. megaterium* expression system and purified by Ni^2+^ affinity chromatography.

To investigate whether the deletion causes improper folding of the proteins, we performed CD spectral analysis and estimated elements of the secondary structure of the toxins on the basis of the CONTIN algorithm. This showed that the mutant toxins maintained a similar secondary structure to TcdB_fl_ (Table 1). The decrease in the alpha helical structure in the composition of TcdB_∆1756–1780_ may be due to the deletion of AA 1756–1780.

### 2.2. Cytopathic and Cytotoxic Effects of TcdB_Δ1756–1780_

**Figure 2:**
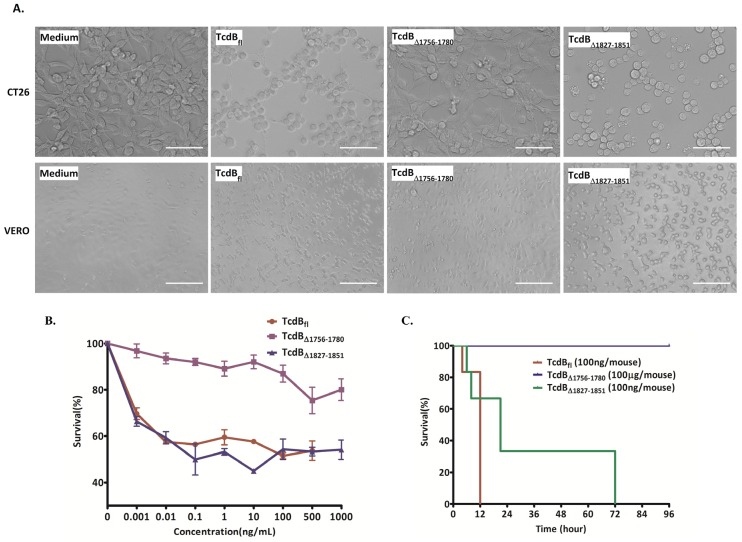
In vitro/in vivo toxicity of TcdB_fl_, TcdB_∆1756–1780_, and TcdB_∆1827–1851_. (**A**) Cytopathic effects induced by TcdB_fl_, TcdB_∆1756–1780_, and TcdB_∆1827–1851_. CT26 and Vero cell lines were incubated with TcdB_fl_ (1 pg/mL), TcdB_∆1756–1780_ (1 μg/mL), and TcdB_∆1827–1851_ (1 pg/mL) for 24 h before microscopic analysis of cell morphology, scale bar = 50 µm; (**B**) Comparison of cytotoxicity of TcdB_∆1756–1780_ and TcdB_∆1827–1851_ with TcdB_fl_. The CT26 cell line was incubated with the indicated concentrations of toxins for 72 h and the viability of the cells was measured by MTT assay. Cells without exposure to toxin were set to show 100% survival; (**C**) In vivo toxicity of TcdB_fl_, TcdB_∆1756–1780_, and TcdB_∆1827–1851_. Groups of mice (*n* = 6) were injected intraperitoneally with TcdB_fl_ (100 ng/mouse), TcdB_∆1756–1780_ (100 μg/mouse), or TcdB_∆1827–1851_ (100 ng/mouse). The survival of the mice was monitored for 96 h.

The cytopathic effects of TcdB_fl_, TcdB_∆1756–1780_, and TcdB_∆1827–1851_ on CT26 and Vero cell lines were compared using a cell rounding experiment. As shown in Figure 2A, both TcdB_fl_ and TcdB_∆1827–1851_ caused 100% cell rounding at a concentration of 1 pg/mL, while no obvious cell rounding was observed after treatment with 1 μg/mL TcdB_∆1756–1780_ for 24 h. Furthermore, the result of the MTT assay indicated that the cytotoxic activity of TcdB_∆1756–1780_ was decreased by about 6–7 logs compared with TcdB_fl_ and TcdB_∆1827–1851_ (Figure 2B). Finally, the in vivo toxicity of the toxins was examined by challenging BALB/C mice, and the survival of the mice was observed (Figure 2C). Mice challenged with TcdB_fl_ (100 ng/mouse) died within 12 h, while mice challenged with TcdB_∆1827–1851_ (100 ng/mouse) died within 72 h. By contrast, mice challenged with TcdB_∆1756–1780_ (100 μg/mouse), a concentration 1000-fold higher than that of TcdB_fl_ and TcdB_∆1827–1851_, showed no signs of disease and survived until the end of observation (96 h). These results demonstrated that the alpha-helix located at AA 1756–1780 is the critical part of the D97 segment and is essential for the toxicity of TcdB.

### 2.3. Analysis of Cysteine Protease, Glucosyltransferase Activity, and Cellular Binding of TcdB_Δ1756–1780_

**Figure 3:**
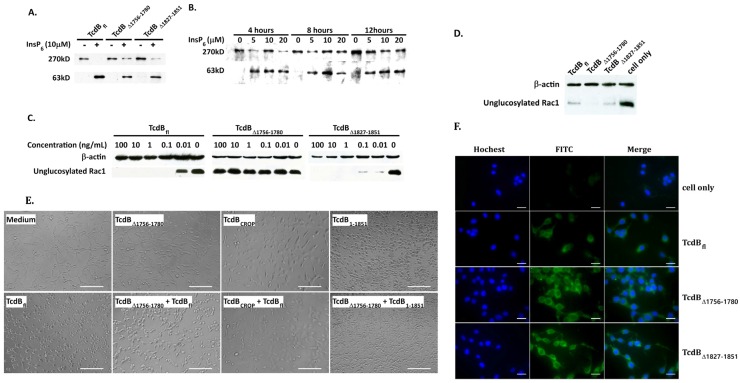
Glucosyltransferase and cysteine protease activities, cellular binding of TcdB_fl_, TcdB_∆1756–1780_, and TcdB_∆1827–1851_. (**A**,**B**). InsP_6_-dependent autocleavage assay. The three toxins were incubated with 10 μM InsP_6_ for 4 h (**A**) and TcdB_∆1756–1780_ was incubated with a series of concentrations of 5, 10, and 20 μM InsP_6_ for a time course of 4, 8, and 12 h (**B**). Western blotting was used to analyze the autocleavage of toxins using anti-TcdB_GTD_ antiserum. (**C**) Intact CT26 cells were exposed to a series of concentrations of 0.01, 0.1, 1.0, 10, 100 ng/mL TcdB_fl_, TcdB_Δ1756–1780_, and TcdB_Δ1827–1851_ respectively for 4 h before western blot analysis using anti-unglucosylated Rac1 monoclonal antibody. β-actin was used as an equal loading control. (**D**) CT26 cells were lysed and then centrifuged. The supernatant was incubated with 100 μg/mL TcdB_fl_, TcdB_Δ1756–1780_, and TcdB_Δ1827–1851_ respectively for 4 h, and the samples were analyzed by western blot. (**E**) Competition experiments. Microscopic images of CT26 cells that were treated for 2 h at 37 °C with TcdB_fl_ (100 pM), TcdB_∆1756–1780_ (250 nM), TcdB_CROP_ (500 nΜ), and TcdB_1-1851_ (0.5 nM) respectively, or with TcdB_fl_ (100 pM)/TcdB_1-1851_ (0.5 nM) together with either TcdB_∆1756–1780_ (250 nM) or TcdB_CROP_ (500 nΜ). Scale bar = 100 µm. (**F**) CT26 cells grown on coverslips were fixed with 4% paraformaldehyde and then incubated with 50 μg of FITC-labeled toxins (FITC-TcdB_fl_ FITC-TcdB_∆1756–1780_ and FITC-TcdB_Δ1827–1851_) respectively for 30 min at 4 °C, before the cells were washed with PBS. The cells were imaged by fluorescence microscopy. Scale bar = 25 µm.

To determine whether the deletion of AA 1756–1780 influenced the structure and function of CPD, GTD, and RBD, their cysteine protease activity, glucosyltransferase activity, and cellular binding were examined. Autoprocessing by the CPD was tested by an in vitro autocleavage assay. As shown in Figure 3A,B, both TcdB_∆1756–1780_ and TcdB_fl_ successfully induced autocleavage, releasing a 63kD fragment containing GTD, in the presence of 10 μM InsP_6_. Furthermore, the concentration dependence of InsP_6_ and the time course of the cleavage reaction of TcdB_Δ1756–1780_ were investigated. This showed that TcdB_Δ1756–1780_ underwent autocleavage after incubation with a series of concentrations of InsP_6_ for 4, 8, and 12 h. Subsequently, CT26 cell lysate and intact cells were used as substrate, respectively, to check the glucosyltransferase activity of TcdB_Δ1756–1780_. The TcdB_Δ1756–1780_ efficiently induced Rac1 glucosylation using CT26 cell lysate as the substrate (Figure 3D). However, TcdB_∆1756–1780_ failed to glucosylate Rac1 in intact CT26 cells (Figure 3C). According to the results above, we concluded that the deletion of amino acids 1756–1780 does not change the structure and function of the cysteine protease and glucosytransferase domains, but speculated that it may result in an inability to deliver GTD into the host cytosol.

Failure of cellular binding, uptake, and translocation may subsequently lead to unsuccessful toxin delivery. The cell surface binding of the mutant toxin was explored using a competition experiment. CT26 cells were incubated with 100 pM TcdB_fl_ in the presence or absence of TcdB_∆1756–1780_ or TcdB_CROP_ (the RBD of TcdB, AA 1852–2366) for 2 h. As observed microscopically (Figure 3E), a 2500-fold concentration of TcdB_∆1756–1780_ competitively inhibited the toxic effect of TcdB_fl_ at a similar level to 5000-fold TcdB_CROP_. It seemed that the competitive ability of TcdB_∆1756–1780_ was two-fold stronger than that of TcdB_CROP_, which is perhaps due to the existence of a second CROP-independent receptor-binding site and the deletion of region 1756–1780 does not change the binding ability of this CROP-independent binding site. For further confirmation of binding ability of TcdB_∆1756–1780_, CT26 cells were incubated with FITC labeled toxins on ice and imaged by fluorescence microscopy. As shown in Figure 3F, TcdB_fl_, TcdB_∆1756–1780_, and TcdB_Δ1827–1851_ were able to bind to the surface of CT26 cells. These results demonstrated that TcdB_∆1756–1780_ maintains approximately the same cellular binding activity as full length TcdB.

### 2.4. Conformational Change at Low pH and Pore Formation by TcdB_∆1756–1780_

**Figure 4:**
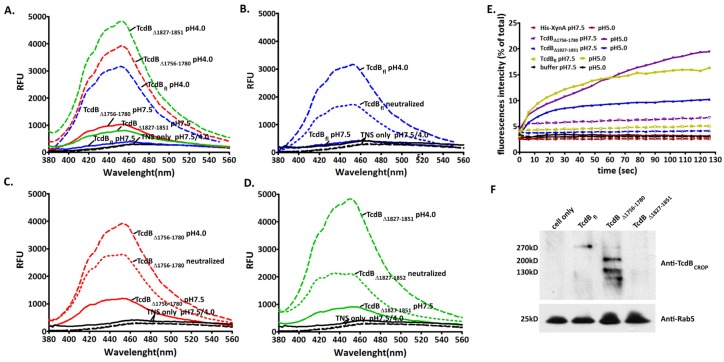
Conformational changes, pore formation, and behavior of the toxins in endosomes. (**A**–**D**) Conformational changes of the toxins under low pH using TNS fluorescence assay. 1 μg of TcdB_fl_, TcdB_∆1756–1780_, and TcdB_∆1827–1851_ were diluted in 150 μM TNS solution at pH 4.0 or pH 7.5 (**A**). In the neutralization experiment, 8 μL of 1 M NaOH solution was added to 1 μg of TcdB_fl_ (**B**), TcdB_∆1756–1780_ (**C**), and TcdB_∆1827–1851_ (**D**) reaction mixtures (pH 4.0) to assess refolding of the toxins. The increase of fluorescence induced by the binding of TNS to the hydrophobic region of the toxins was measured by SpectraMax M5. E. Pore formation by toxins in HPTS/DPX loaded LUVs. The pore formation was induced by acidic conditions. The fluorescence intensity was monitored on a SpectraMax M5 (Molecular Devices, Sunnyvale, CA, USA) with an excitation of 454 nm and an emission of 520 nm. Total HPTS fluorescence was determined by addition of Triton X-100 (to 0.3%), and His-XynA was used as the negative control. F. Endosome isolation and anti-TcdB western blot. CHO cells were exposed to 2 μg/mL of toxins at 37 °C for 2 h before harvesting. The endosome compartments were isolated by immunoadsorption using anti-Rab5 monoclonal antibody. Subsequently, the isolated endosomes were analyzed by western blot using anti-TcdB_CROP_ antisera.

pH-induced changes in toxin hydrophobicity were identified by the TNS assay, which is a convenient probe for determining the exposure of hydrophobic domains under various conditions. As shown in Figure 4A, both TcdB_fl_ and TcdB_Δ1756–1780_ exhibited a dramatic increase in TNS-associated fluorescence at pH 4.0. Additionally, when the buffer was neutralized by 1 M NaOH, the fluorescence of both toxins decreased (Figure 4B–D).

Exposure of the hydrophobic region of the toxins enabled membrane insertion, which is paralleled by pore formation, resulting in the entrance of toxins into host cells directly through the endosomal membrane. The pore formation of TcdB_Δ1756–1780_ was studied by assay of fluorophore leakage from HPTS/DPX loaded LUVs. Pore formation was monitored by the release of the fluorescent dye HPTS, which is quenched in the lipid vesicles by DPX. The HPTS/DPX compounds were released and diluted when pores formed in the LUVs, which caused an increase in fluorescence by reduction of collision quenching. As shown in Figure 4E, under low pH conditions, TcdB_fl_ and TcdB_Δ1756–1780_ induced pore formation in LUVs and caused an increase in fluorescence. However, no increase in the fluorescent signal was observed under acidic pH with protein His_6_-XynA, which was used as a negative control. Comparing TcdB_Δ1756–1780_ with TcdB_fl_, the fluorescence of TcdB_fl_ increased slightly faster than that of TcdB_Δ1756–1780_, but this may not be significant because they displayed a similar pattern of increase in general (Figure 4E). From all these results, we concluded that the mutant toxin TcdB_Δ1756–1780_ induced conformational change and pore formation under acidic pH conditions in a similar pattern to TcdB_fl_. However, there is insufficient information about the exact mechanism by which these processes take place. We speculated that the deletion of region 1756–1780 led to locking of the TcdB in endosomes, resulting in a failure to deliver GTD.

### 2.5. Behavior in Endosomes

Endosome isolation was performed and western blot analysis was used to obtain further insight into the behavior of the toxins in endosomes. Rab5 is a regulatory guanosine triphosphatase that has been localized to the plasma membrane, clathrin-coated vesicles, and early endosomes. It participates in endosomal membrane fusion reactions and is important in control of endocytic function [24,25]. Rab5 is often used as an early endosome marker [24]. In this study, endosome populations were purified by immunoadsorption from syringe-lysed cell lysate using an antibody against Rab5 protein and were analyzed by western blot using anti-TcdB antibodies. The result showed (Figure 4F) that holotoxin of TcdB_fl_ and TcdB_Δ1827–1851_ can be recognized by anti-TcdB_CROP_ antibody, which is specific for the receptor binding domain of TcdB, but the band of TcdB_Δ1827–1851_ was much weaker than that of TcdB_fl_. In contrast, a very small amount the holotoxin of TcdB_Δ1756–1780_ was detected as well as several bands of lower molecular weight. Based on these results, we hypothesize that the majority of TcdB_Δ1756–1780_ is rapidly degraded in the endosome, which prevents efficient delivery of GTD into the cytosol. Therefore, a much higher concentration of TcdB_Δ1756–1780_ would be needed to induce the same level of cell rounding or cell death when compared with wild type TcdB.

## 3. Discussion

The goal of this study is to identify the critical part of D97 segment and provide insight into the structure-function relationship of the TcdB translocation action. Over decades, efforts have been made to reveal the mechanism of delivery of the cytotoxic glucosyltransferase domain across the endosomal membrane into cytosol. Despite significant advances, there is still a long way to completely reveal the underlying mechanism of the toxin delivery. In 2013, we found that a 97-amino-acid segment (D97) located in the *C*-terminus of the translocation domain is essential for the toxicity of TcdB, and the D97 segment was hypothesized to be involved in the translocation of TcdB because TcdB-D97 failed to release GTD into the host cytosol [23]. In this study, we narrowed down the critical region of D97 and tried to investigate the function of this region. Using algorithms GOR4, SIMPA96, and Chou-Fasman (ProtScal), the secondary structure of D97 segment was predicted, demonstrating that the region covering AA 1756–1780 formed an alpha helical structure and it was hypothesized to be important. Therefore, a TcdB deletion mutant TcdB_∆1756__–1780_ was constructed, while TcdB_∆1827–1851_ was used as a control protein. TcdB_∆1827–1851_ still exhibited almost complete in vitro and in vivo toxicity when compared with TcdB_fl_, however, toxicity of TcdB_∆1756–1780_ had dramatically decreased by 6–7 logs. To this point, it is inferred that the region 1756–1780 plays an important role in D97, while the region 1781–1851 may not be essential for the toxicity of TcdB. To investigate the function of the region 1756–1780, the glucosyltransferase and cysteine protease activities, cellular binding, pH-dependent conformational change and pore formation of TcdB_∆1756–1780_ were examined. As results, TcdB_∆1756–1780_ maintains approximately intact functions of the GTD, CPD, and RBD as TcdB_fl_. However, TcdB_∆1756–1780_ was able to induce Rac1 glucosylation only when cell lysate was used as the substrate but failed when using intact cells as substrate. It is hypothesized that the deletion of 25AA 1756–1780 impairs the function of the TD. Previous studies showed that the translocation process of *C. difficile* TcdB is associated with pH-induced conformational change and pore formation. Therefore, pH-induced conformational change and pore formation were studied by TNS fluorescence analysis and the LUVs fluorophore leakage assay respectively. The results indicated that TcdB_∆1756–1780_ had a similar pattern of conformation change and pore formation to TcdB_fl_ under acidic conditions, which would suggest similar function in endosomes. According to our previous report, TcdB-D97 was concluded to be trapped in endosomes. Therefore, we investigated the differences between TcdB_fl_ and TcdB_∆1756–1780_ related to the behavior in endosomes. We found that TcdB_∆1756–1780_ was degraded rapidly in endosomes into several fragments with different molecular weights. It is important to note that the region 1756–1780 is only a small part within the translocation domain, the deletion of which may not influence the general trend of conformational change, that is why TcdB_∆1756–1780_ displayed conformational change in similar pattern as TcdB_fl_ in the TNS fluorescence analysis. Nevertheless, the region 1756–1780 may be essential for a critical step in conformational change, and its deletion led to unsuccessful membrane insertion and eventually prevented GTD from being translocated across the endosomal membrane.

Recently, Bjӧrn Schorch et al. [14] proposed a two-receptor model for the cell entry of clostridial glycosylating toxins, in which the CROP domain primarily facilitates the accumulation of the toxins at the cell surface, and the additional receptor-binding domain interacts with another specific cell surface protein to induce the endocytosis of the toxins. A new model of the modular composition of TcdB presented by Selda Genisyuerek et al. [16] suggests that the region between AA 1500 and 1851 is not necessary for translocation and may represent an additional receptor-binding site, as deletion of the region reduced toxicity, but did not complete abrogate it. TcdB_∆1756–1780_ was detected in the endosome, which indicates that it is able to induce endocytosis. Therefore, we suggest that the region 1756–1780 is not necessary for cellular binding but is necessary for efficient GTD translocation. Because of ineffective translocation, after endocytosis, TcdB_∆1756–1780_ was trapped in the endosome and was subsequently degraded into small fragments. The majority of the intact GTD was therefore unable to be translocated into the host cytosol. As a result, a much higher concentration of TcdB_∆1756–1780_ would be needed to trigger same level of cell rounding or cell death as for TcdB_fl_.

**Figure 5:**
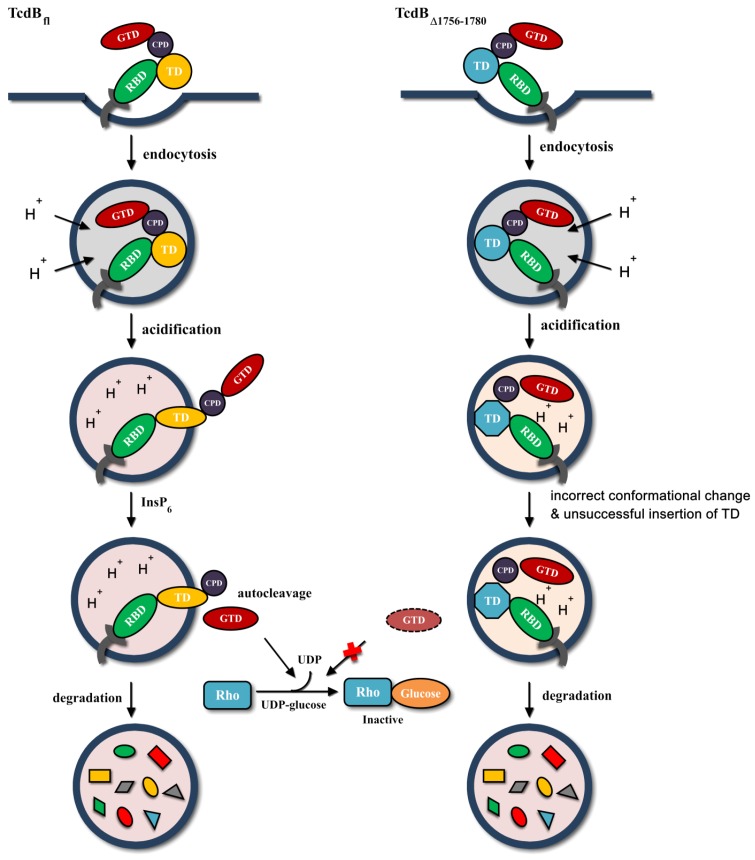
Scheme of TcdB_fl_ and TcdB_∆1756__–1780_ actions of cellular uptake and GTD delivery. Compared to TcdB_fl_, TcdB_∆1756–1780_ proceeds incorrect conformational change and triggers steric hindrance after acidification of endosomes, resulting in unsuccessful membrane insertion and GTD delivery, which eventually leads to toxin trapped and degraded in the endosome compartments.

In summary, we identified a region, 25AA 1756–1780, within D97 that is essential for TcdB toxicity. The region 1756–1780 plays a pivotal role in the translocation of GTD across the endosomal membrane, however, it is not necessary for pore formation and cellular binding. We hypothesize that the region 1756–1780 has a critical role in pH-induced conformational change, and its deletion may lead to an incorrect conformational change, triggering steric hindrance, consequently resulting in unsuccessful membrane insertion and GTD delivery. Eventually, deletion of region 1756–1780 leads to toxin trapped and degraded in the endosome compartment (Figure 5). Determination of the crystal structure and further study of the translocation domain are needed to explain the function of the 1756–1780 region and to clarify the mechanism of action involved in TcdB translocation.

## 4. Materials and Methods

### 4.1. Mammalian Cell Lines

CT26 cells (BALB/C mouse colon tumor cells), Vero cells (kidney epithelial cells from African green monkeys), and CHO cells (Chinese hamster ovary cells), purchased from the Chinese Academy of Sciences Institute of Cell Resource Center, were cultured in Dulbecco’s minimum Eagle’s medium supplemented with 10% fetal bovine serum, 100 units/mL penicillin, and 100 μg/mL streptomycin in 100 mm culture plates at 37 °C and 5% CO_2_.

### 4.2. Bacteria Strains

*Bacullus megaterium* WH320 (MoBiTec, Goettingen, Germany) and *Escherichia coli* DH5α (Takara, Kyoto, Japan) were cultured in LB medium at 37 °C unless otherwise indicated.

### 4.3. Cloning of TcdB Constructs

DNA corresponding to the TcdB fragments (amino acids 1–1755, 1–1826, 1–1851, 1781–2366, and 1852–2366) was amplified from cDNA encoding to full length TcdB by PCR. Subsequently, TcdB constructs with internal deletions (TcdB_Δ1756–1780_ and TcdB_Δ1827–1851_) were generated by overlap PCR using the PCR products of TcdB sequences encoding amino acids 1–1755 and 1781–2366, and 1–1826 and 1852–2366 as templates, respectively. The overlap PCR products and the fragments 1–1851 were cloned into the pHis1525 (MoBiTec, Goettingen, Germany) vector using the restriction sites *Bsr*GI and *Kpn*I.

### 4.4. Protein Expression and Purification

The transformation of *B. megaterium* protoplast was performed according to the manufacturer’s instructions (MoBiTec, Goettingen, Germany). The transformed *B. megaterium* colonies were transferred to the LB broth medium containing 10 μg/mL tetracycline and incubated overnight at 37 °C with 250 rpm. The overnight culture was diluted 1:100 in LB broth medium containing tetracycline and grown to an optical density OD_600_ around 0.3 before the addition of xylose (5 mg/mL) to induce protein expression.

All proteins were expressed with *C*-terminal His_6_ tags. The purification of His-tag proteins was performed by Ni^2+^ affinity chromatography. Briefly, the *B. megaterium* pellet was suspended in 5 mL lysis buffer (20 mM phosphate sodium buffer, 500 mM NaCl, 30 mM imidazole, pH 7.4) per 100 mL bacterial culture. Cells were disrupted by sonication and the lysate was centrifuged at 15,500× *g* for 30 min at 4 °C. The supernatant was applied to a nickel-charged HisTrap HP column (GE Healthcare Bio-Sciences, Pittsburgh, PA, USA) and the bound protein was eluted using elution buffer (20 mM phosphate buffer, 500 mM NaCl, 500 mM imidazole, pH 7.4). The proteins were dialyzed to PBS buffer containing 20% glycerol and stored at −80 °C.

Circular dichroism (CD) spectrophotometry was performed using a Chirascan Circular Dichroism Spectropolarimeter (Applied Photophysis Limited, Surrey, UK), with a scan interval of 1 nm and path length of 0.1 cm at 25 °C. The CD data were analyzed on the basis of the CONTIN algorithm.

### 4.5. Cytotoxic and Cytopathic Effects

The cytotoxic effect induced by the toxins was analyzed by MTT (Methylthiazolyldiphenyl—tetrazolium bromide) viability assay as described previously [26]. Briefly, 2 × 10^4^ CT26 cells were seeded in a 96-well plate and cultured at 37 °C for 24 h. Serial dilutions of each toxin were added to the cells and incubated at 37 °C for another 72 h. Following this, 10 μL of MTT was added and the plate was further incubated at 37 °C for 2 h. The formazan was solubilized with DMSO, and absorbance at 570 nm was measured using a SpectraMax M5 (Molecular Devices, Sunnyvale, CA, USA). Cell viability was expressed as a percentage of the cell survival in the control wells.

The cytopathic effect was measured by a cell rounding experiment. CT26 or Vero cells were incubated with TcdB_fl_ (full length TcdB), TcdB_Δ1756–1780_, and TcdB_Δ1827–1851_ at indicated concentrations for 24 h and cell rounding was observed by light microscopy. The experiments were repeated three times, and triplicate wells were assessed for the MTT assay and cell rounding in each experiment.

### 4.6. Mouse Systemic Toxin Challenge

6- to 8-week-old BALB/C mice (SPF) were purchased from Guangdong Medical Laboratory Animal Center (Guangdong, China). Groups of mice (*n* = 6) were challenged intraperitoneally with TcdB_fl_ (100 ng/mouse), TcdB_Δ1756–1780_ (100 μg/mouse), and TcdB_Δ1827–1851_ (100 ng/mouse), respectively. Mouse survival was monitored every six hours. The animal protocols used in this work were approved by Guangdong Provincial Department of Science and Technology (Approval Number: SYXK (Yue) 2014-0145). This research does not violate any national guidelines and institutional policies for use of animal in research.

### 4.7. In Vitro Autocleavage Assay

The in vitro autocleavage assay was performed as described previously [6,27]. Each toxin protein was diluted in 20 mM Tris buffer (pH 7.4) in a final volume of 100 μL. Cleavage was initiated by addition of 10 μM inositol hexakisphosphate (InsP_6_) (Sigma, St. Louis, MO, USA) and the mixture was incubated at 37 °C for 12 h. To investigate the dependence of the effect on the time course and the concentration of InsP_6_, the toxins were incubated with 5, 10, and 20 μM InsP_6_ for 4, 8, and 12 h, respectively. The reaction was stopped by SDS-PAGE sample loading buffer, and analyzed by western blot using anti-TcdB_GTD_ (the GTD of TcdB, amino acids 1–543) antiserum prepared by our laboratory.

### 4.8. In Vitro Glucosylation Assay

The in vitro glucosylation assay was performed with intact CT26 cells and cell lysates. In the experiment with intact cells, 2 × 10^5^ CT26 cells were seeded in a 24-well plate and cultured at 37 °C in 5% CO_2_ for 36 h before being treated with different concentrations of TcdB_fl_, TcdB_Δ1756–1780_, and TcdB_Δ1827–1851_ respectively for 4 h. After treatment, the cells were washed with PBS three times, then lysed by SDS-PAGE sample loading buffer and boiled for 5 min. In the cell lysate experiment, CT26 cell pellets were resuspended in a reaction buffer (50 mM HEPES pH 7.5, 100 mM KCl, 1 mM MnCl_2_, and 2 mM MgCl_2_) and lysed by passing through a 30 G needle 40 times. After centrifugation, the supernatant was used as the cytosolic fraction. For the glucosylation assay, the cytosolic fraction was incubated with 100 μg/mL TcdB_fl_ or TcdB_Δ1756–1780_ at 37 °C for 4 h. The reaction was terminated by adding SDS-PAGE sample loading buffer and was boiled for 5 min. Finally, the samples were analyzed by anti-unglucosylated Rac1 mAb (BD Biosciences, San Diego, CA, USA) using western blot analysis.

### 4.9. Analysis of Cell Surface Binding of the Toxins

In the competition experiment, CT26 cells were incubated with 10 pM TcdB_fl_ for 3 h in the presence or absence of 250 μM TcdB_Δ1756–1780_, or 500 μM TcdB_CROP_ (the RBD of TcdB, amino acids 1852-2366). Meanwhile, CT26 cells were incubated with 0.5 nM TcdB_1-1851_ (the fragment without CROP, amino acids 1-1851) for 4 h with or without 250 µM TcdB_Δ1756–1780_. Subsequently, the morphology of the CT26 cells was visualized by light microscopy.

In the immunofluorescence experiment, CT26 cells grown on coverslips to 60% confluence were fixed with 4% paraformaldehyde and subsequently incubated with 50 μg of the FITC-toxins (FITC-TcdB_fl_, FITC-TcdB_∆1756–1780_, and FITC-TcdB_Δ1827–1851_) respectively for 30 min at 4 °C. The cells were then washed with PBS and imaged by fluorescence microscopy.

### 4.10. TNS Fluorescence Analysis of Conformational Change of Toxins

The pH-induced conformational change in the toxins was examined as described previously [18]. Briefly, 2-(*p*-toluidinyl) naphthalene-6-sulfonic acid, sodium salt (TNS, Sigma, St. Louis, MO, USA) was dissolved in pH 4.0 or pH 7.5 buffer (100 mM NaCl, 100 mM ammonium acetate, 1 mM EDTA) to a concentration of 150 μM. Following this, 1 μg of TcdB_fl_, TcdB_Δ1756–1780_, and TcdB_Δ1827–1851_ were mixed into 100 μL TNS solution and incubated at 37 °C for 30 min. In the neutralization experiment, 8 μL of 1 M NaOH solution was added to the pH 4.0 reaction mixtures to assess refolding of the toxins. The samples were analyzed by SpectraMax M5 (Molecular Devices, Sunnyvale, CA, USA) with an excitation of 366 nm and an emission scan of 380–500 nm.

### 4.11. Fluorophore Leakage from Lipid Vesicles

Pore formation by the toxins was measured by the fluorophore leakage from large unilamella lipid vesicles (LUVs), as previously described [16]. Briefly, the LUVs were prepared with egg phosphatidylcholine (EPC, Sigma, St. Louis, MO, USA) and cholesterol (Chol, Sigma, St. Louis, MO, USA) in a molar ratio of EPC/Chol 3:1 and 0.8% 1,2-dioleoyl-sn-glycero-3-[(*N*-(5-amino-1-carboxypentyl)iminodiacetic acid)succinyl] (nickel salt) (DGS-NTA[Ni]) (Avanti Polar Lipids, Inc., Alabaster, AL, USA). The LUVs loaded with the fluorophore mixture of 8-hydroxypyrene-1,3,6-trisulphonic acid (HPTS, Sigma, St. Louis, MO, USA) and p-xylenebis(*N*-pyridinium bromide) (DPX, Sigma, St. Louis, MO, USA) were prepared according to the method described by Peschka-Sϋss and Schubert in 2003 [28]. In the leakage assay, the LUVs were diluted to an appropriate concentration in 20 mM Tris buffer (pH 7.4 or 5.0) before toxin addition. The fluorescence of the mixture was monitored on a SpectraMax M5 (Molecular Devices, Sunnyvale, CA, USA) with an excitation of 454 nm and an emission of 520 nm. Total HPTS fluorescence was determined by addition of Triton X-100 (to 0.3%), and His_6_-XynA was used as a negative control.

### 4.12. Endosome Isolation

The CHO cells were seeded in a 100 mm culture dish and incubated at 37 °C for 48 h. After exposure to 2 μg/mL of toxins at 37 °C for 2 h, the cells were scraped off in 1 mL PBS buffer with a plastic spreader and pelleted in a 1.5 mL tube (750× *g*, 2 min centrifugation). The cell pellet was suspended in 500 μL PBS and lysed by passing through a 30 G syringe 20–30 times until the cells were broken but the nuclei were left intact as seen by light microscopy. Subsequently, the cell lysate was centrifuged at 500× *g* and 4 °C for 2 min and the supernatant was applied to immunoadsorption against the early endosome marker Rab5.

Goat anti-rabbit IgG coated magnetic beads (Dynabeads M-280) were purchased from Invitrogen (ThermoFisher Scientific, Waltham, MA, USA). Following the manufacturer’s instructions, the beads were washed with washing buffer (20 mM PBS, 0.1% BSA, 2 mM EDTA, pH 7.4) before incubation with primary antibodies (anti-Rab5 mAb from rabbits, Abcam, Cambridge, MA, USA) at 4 °C for 12 h. For immunoadsorption, the beads coated with primary antibody were incubated with the cell lysate at 4 °C for 12 h. Subsequently, the beads were collected with a magnet (ThermoFisher Scientific, Waltham, MA, USA) and washed three times with washing buffer (20 mM PBS, 2 mM EDTA, 1% BSA, pH 7.4). The samples were then analyzed by western blot.

## Figures and Tables

**Table 1:** Summary of secondary structure elements calculated from CD data.

Protein	Α-helix (%)	β-sheet (%)	Random Coil (%)	Total (%)
rTcdB_fl_	6.5	52.6	40.8	99
TcdB_Δ1756-1780_	5.8	53.5	40.9	100.2
TcdB_Δ1827-1851_	6.1	53.1	40.8	100
