# Correction: Chen, S., et al. Identification of an Essential Region for Translocation of *Clostridium difficile* Toxin B. *Toxins* 2016, *8*, 241

**DOI:** 10.3390/toxins8120352

**Published:** 2016-12-02

**Authors:** Shuyi Chen, Haiying Wang, Huawei Gu, Chunli Sun, Shan Li, Hanping Feng, Jufang Wang

**Affiliations:** 1 School of Bioscience and Bioengineering, South China University of Technology, Guangzhou 510006, China; shuyichan@foxmail.com (S.C.); yingzi224926@163.com (H.W.); guhuawei1990@163.com (H.G.); chunlis@163.com (C.S.); lishan@scut.edu.cn (S.L.); 2 Department of Microbial Pathogenesis, University of Maryland Dental School, Baltimore, MD 21201, USA; hfeng@umaryland.edu

The authors wish to make the following corrections to their paper [1].

In the caption of Figure 3, “and TcdB_1-1851_ (0.5 nM) respectively” should be replaced with “respectively, and treated for 4 h with TcdB_1-1851_ (0.5 nM)”.

In the first paragraph of Section 4.9, “CT26 cells were incubated with 10 pM TcdB_fl_ for 3 h in the presence or absence of 250 μM TcdB_Δ1756–1780_, or 500 μM TcdB_CROP_” should be replaced with “CT26 cells were incubated with 100 pM TcdB_fl_ for 2 h in the presence or absence of 250 nM TcdB_Δ1756–1780_, or 500 nM TcdB_CROP_”. Also, “for 4 h with or without 250 µM TcdB_Δ1756–1780_” should be replaced with “for 4 h with or without 250 nM TcdB_Δ1756–1780_”.

The changes do not affect the scientific results. The manuscript will be updated and the original will remain online on the article webpage. We apologize for any inconvenience caused to our readers.
